# Effects of preoperative melatonin on postoperative pain following cesarean section: A randomized clinical trial^☆^^☆☆^^☆☆☆^

**DOI:** 10.1016/j.amsu.2021.102345

**Published:** 2021-05-12

**Authors:** Farshad Hassanzadeh Kiabi, Seyed Abdollah Emadi, Aghdas Ebadi Jamkhaneh, Goli Aezzi, Negar Shahzadeh Ahmadi

**Affiliations:** a Department of Anesthesiology, School of Medicine, Mazandaran University of Medical Sciences, Sari, Iran; b Department of Obstetrics and Gynecology., School of Medicine, Mazandaran University of Medical Sciences, Sari, Iran; c Student of Research Committee, Mazandaran University of Medical Sciences, Sari, Iran

**Keywords:** Melatonin, Surgery, Analgesia, Cesarean, Pain, Spinal anesthesia

## Abstract

**Background:**

Data regarding the analgesic effects of melatonin after the surgery is scare. So far, only one study has investigated the analgesic effect of melatonin during cesarean section. **Objective**: The present study was performed to investigate the effect of preoperative oral melatonin on pain intensity after cesarean section during spinal anesthesia.

**Methods:**

In a double-blind randomized controlled clinical trial study, 204 patients undergoing elective cesarean section with class 1 and 2 anesthesia (ASA) were enrolled. Patients were randomly divided into 3 groups of 68 patients. Patients in group A were given 5 mg melatonin tablets, patients in group B were given 10 mg melatonin tablets, and group C was given placebo. All patients underwent spinal anesthesia with the same anesthesia protocol. Pain intensity, nausea, vomiting, pruritus and headache were assessed and recorded 2, 6, 12 and 24 h after surgery. The time of first dose of analgesia, the amount of opioid consumed within 24 h, and the time to resume physical activity was also recorded. Statistical analysis of data was performed using SPSS 20 software.

**Results:**

Repeated measurements of pain intensity during the study showed that in all 3 groups pain intensity was significantly reduced during the study, p < 0.001, respectively. The intensity of pain was significantly different in groups B and C, groups B and A and groups A and C, P < 0.001, respectively. The pain reduction was greatest in group B, followed by group A and group C, respectively. The time interval between the end of surgery and the patient's need for analgesia was significant in group B compared to group A (P = 0.035) and C (P < 0.001) and also in group A compared to group C (P = 0.011). The mean dose of opioid was significantly least in group B, p < 0.001. The mean time to resume physical activity was also shortest in group B, p < 0.001 Headache and nausea/vomiting were observed in 7 patients (10.7%) group A and 7 patients (10.7%) in group C. None of the patients in group B developed complications.

**Conclusion:**

The results of the present study showed that the use of 10 mg of melatonin before cesarean section with spinal anesthesia is not only safe, but also reduces the severity of patients' pain, increases the duration of postoperative analgesia, reduces the need for analgesics after surgery and resumption of physical activity.

## Highlights

• Data regarding the analgesic effects of melatonin after the surgery is scare.
• So far, only one study has investigated the analgesic effect of melatonin during cesarean section.
• 10 mg of melatonin before cesarean section with spinal anesthesia is not only safe.
• Reduces the severity of patients' pain, increases the duration of postoperative analgesia.
• Reduces the need for analgesics after surgery and resumption of physical activity.

## 1 Introduction

Postoperative pain has been a major concern after surgery that impairs patient's ability to resume back to daily-life activity, imposes psychological effects and increases the requirement of analgesic like opioids, that can have side-effects and can lead to addition [1]. It is reported that approximately 75–80% of patients experience moderate to severe postoperative pain even after receiving analgesic treatment [2,3]. Cesarean section is the most common gynecological procedure where its incidence is reported to be 40–50% in public hospital [4]. Pain following cesarean section is presented with an added challenge [5], since it impairs mothers' ability to look after the newborn [6]. On the other hand, pain relief after cesarean section is very important due to the increased risk of thromboembolic diseases that may be exacerbated by inactivity due to postoperative pain [7]. The consequences of these complications include various economic and medical problems such as increasing the length of hospital stay, the need for readmission, increasing the costs of patient recovery and finally patients' dissatisfaction with hospital care [8,9].

Conventionally, for the management of acute postoperative pain, mainly oral or injectable (intramuscular and intravenous) analgesics are administered [10]. Narcotic drugs, especially in injectable form, is beneficial in relieving acute pain [11,12]. However, opioid use is associated with dose-dependent complications such as respiratory depression, nausea, vomiting, urinary retention, pruritus, drowsiness, or postoperative ileus [13,14]. Therefore, it seems reasonable to use compounds that can intensify the analgesic effects of narcotics and thus create better analgesic effects with less opioid use [15,16].

Melatonin, or *N*-acetylmethoxytryptamine, is a hormone secreted by the pineal gland in the brain. Light is the main factor in the environment that regulates melatonin production. Melatonin has important biological effects on the body and plays an important role in regulating the sleep-wake cycle [17,18]. Studies have shown the protective role of melatonin in various diseases such as cancer, cardiovascular disease, Alzheimer's, diabetes, mood disorders, gastrointestinal diseases, fibromyalgia and mental disorders [19,20]. Findings of studies have shown that anesthesia and surgery impair the secretion of melatonin from the pineal gland [21]. Another study of patients undergoing orthopedic surgery showed that anesthesia with surgery significantly reduced the amount of melatonin sulfatoxy (one of the most important metabolites of melatonin) in the evening of the first day after surgery and anesthesia [22]. Melatonin supplementation has been recommended in patients undergoing surgery [23]. Contradictory results have been obtained in various clinical studies investigating the analgesic effects of melatonin after surgery, which have used different doses of melatonin (including 3, 5, 6 and 10 mg) [[24], [25], [26], [27]].

We designed a randomized double blinded clinical study to evaluate the analgesic effects of preoperative 5 mg and 10 mg melatonin among cesarean section patients referred to our center.

## 2 Methods

In this double blinded clinical trial, women referred to (XXX) for elective cesarean section from January 2019–December 2019 with American Society of Anesthesiologists (ASA) classification I and II were included. Inclusion criteria was: the patient's desire to participate in the study via consent, candidate for non-emergency cesarean section, insensitivity to melatonin, age between 40 and 20 years, meeting ASAI-II criteria, cecond cesarean section (CS II), no history of seizures, preeclampsia, eclampsia, hypertension and organ transplantation, no use narcotic painkillers for 24 h before the intervention, no abuse alcohol or drugs and those patients with term gestational age. The exclusion criteria of the study included: the patient's unwillingness to continue participating in the study, prolongation of cesarean section (more than 1.5 h), increase in the size of the incision for any reason, occurrence of any unusual complication during surgery, failure of spinal anesthesia and its conversion to general anesthesia and patients undergoing third cesarean section (CS III) and above.

The patients were randomly assigned in three groups using codes with Random Allocation Software. Prior to surgery, patients were provided with adequate information and training on how to determine the severity of postoperative pain, nausea, vomiting, pruritus, and headache using the VAS criteria, as well as how to use a PCA (patient-controlled anesthesia) pump. Written consent was obtained from all the patients for the participation in the study.

Since the studies have already reported the insignificance of 3 mg dose of melatonin, the dose of the drug used in the study was 5 mg and 10 mg, respectively. The patients were divided in the three groups, patients in group A received a 5 mg melatonin tablet (made by Nature Made, USA), patients in group B received a 10 mg melatonin tablet., and patients in group C received the placebo 1 h before surgery. Patients and researchers were unaware of group placements, and drugs were coded at the School of Pharmacy at the time of manufacture.

In the operating room, all patients underwent spinal anesthesia with the same anesthesia protocol, which included spinal anesthesia using needle 25 and 2.5 cc of 2.5% Marcaine hyperbaric solution. After surgery and patients entering the ward, a PCA pump was inserted for all patients to control pain. The internal composition of the PCA pump consisted of 25 mg of morphine, 1 g of paracetamol and the rest up to 100 cc (total volume of the PCA pump) of normal saline. The adjustment characteristics of the PCA pump were 0.5 cc bolus and lockout interval of 15 min.

Pain intensity, nausea, vomiting, pruritus and headache in the study groups were evaluated and recorded 2, 6, 12 and 24 h after surgery. In addition, the patient's first request for postoperative analgesia, the patient's opioid intake in the 24 h after surgery, and the time of first bed rest were assessed and recorded. The primary outcomes of the study were the severity of pain and the amount of dose of drugs used and the secondary outcomes were the rate of nausea, vomiting, pruritus and headache. The evaluation was performed by the anesthesia assistant. The placebo required for the study was prepared from starch and the same color and form of melatonin in XXX School of Pharmacy.

All patients entered the study with full knowledge and informed consent. The study was performed after the approval of the ethics committee of (XXX) and also the registration in the International Iranian Clinical Trials Registration Center with IRCT number: **(XXX)**. In addition, patients were assured that all the information in the study would be kept confidential and that researchers would only use it to provide a final report.

After collecting and classifying the data, statistical analysis of the data was performed using SPSS v20 software. Chi-square, T-test, analysis of variance with repeated measures and generalized estimation equations were used for analysis. Also, before examining the primary and secondary outcomes, demographic and clinical features were compared between the three groups. *P*-value less than 0.05 was considered as the significance of the relationship.

This study was approved by the Research Ethics Board of (XXX).

Unique identifying number: researchregistry6527.

The study has been reported in accordance with CONSORT criteria [28].

## 3 Results

**Fig. 1:**
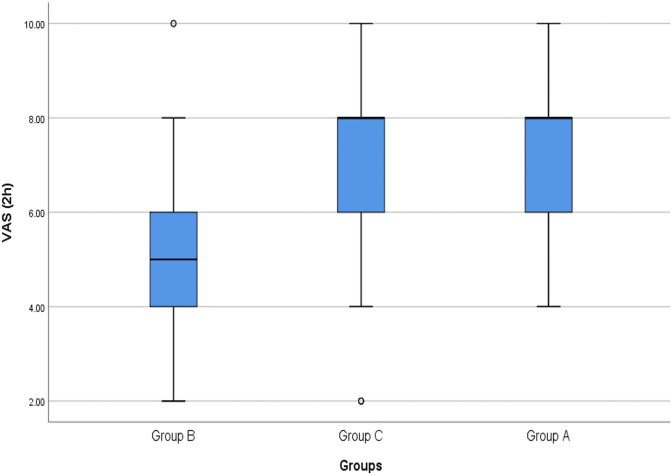
Comparison of mean pain intensity of patients in the study groups 2 h after surgery.

The number of patients in the study was 204 with 68 patients in each group. The mean pain intensity of patients 2 h after the start of the study in group A patients (group receiving 5 mg oral melatonin before surgery) was 7.28 ± 1.40 cm (between 4 and 10, Median = 8 cm), in group B patients (group receiving 10 mg oral melatonin before surgery) was 4.91 ± 1.56 cm (between 2 and 10, Median = 5 cm) and in placebo group (group C) was 7.29 ± 1.82 cm (between 2 and 10, Median = 8 cm) (Fig. 1). There was a statistically significant difference between pain intensity in group A and B patients and pain intensity in group B patients was significantly lower than group A patients (P < 0.001). Also, the mean pain intensity of group B patients was significantly lower than group C patients (P < 0.001). However, pain intensity in group A patients was not statistically significant with group C patients (P = 0.958).

**Fig. 2:**
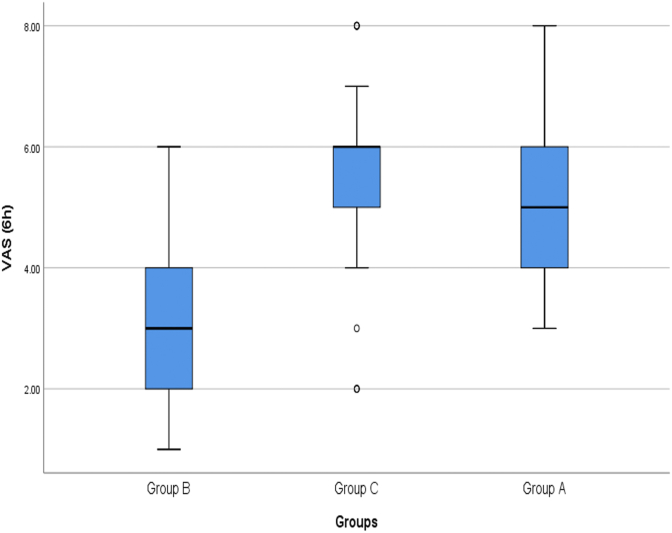
Comparison of mean pain intensity of patients in the study groups 6 h after surgery.

In the study of pain intensity of patients 6 h after surgery, the mean pain intensity in group A was 4.97 ± 1.09 cm (between 3 and 8, Median = 5 cm), in group B was 3.21 ± 1.10 cm (between 1 and 6, Median = 3 cm) and in group C was 5.48. 1.27 cm (between 2 and 8, Median = 6 cm) (Fig. 2). Pain intensity in group B patients was significantly lower than group A patients (P < 0.001) and group C patients (P < 0.001). There was a statistically significant difference between the pain intensity in group A and group C and the pain intensity of group A patients was significantly lower (P = 0.013).

**Fig. 3:**
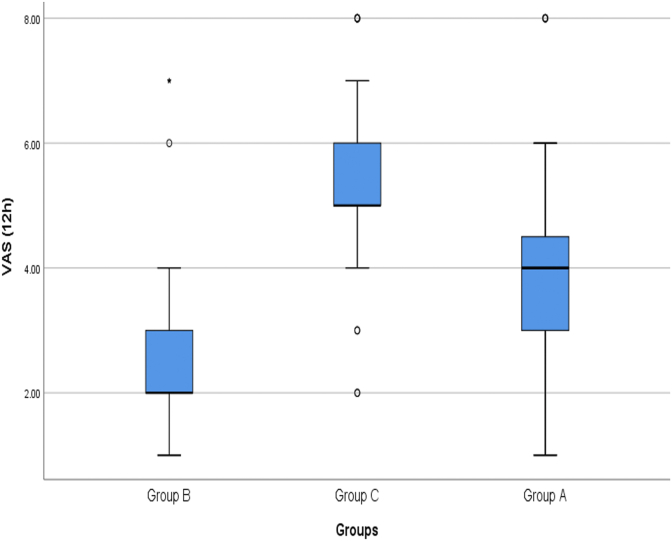
Comparison of mean pain intensity of patients in the study groups 12 h after surgery.

The mean pain intensity of patients 12 h after surgery in group A, B and C patients was 3.85 ± 1.61 cm (between 1 and 8, Median = 4 cm), 2.40 ± 1.24 cm (between 1 and 7, Median = 2 cm) and It was 5.36 ± 1.29 cm (between 2 and 8, Median = 5 cm) (Fig. 3). The mean pain intensity of group B patients was significantly lower than group A patients (P < 0.001) and group C patients (P < 0.001). In addition, pain intensity in group A patients was also significantly lower than group C patients (P < 0.001).

The mean pain intensity 24 h after surgery in group A was 2.59 ± 1.25 cm (between 1 and 6, Median = 2 cm), in group B was 1.50 ± 0.63 cm (between 1 and 3, Median = 1 cm) and in group C was 2.51 ± 1.04 cm (between 1 and 4, Median = 2 cm). Pain intensity of patients in group B, 24 h after surgery was significantly lower than patients in group A (P < 0.001) and group C (P < 0.001). However, there was no statistically significant difference between pain intensity in group A patients and group C patients (P = 0.710).

**Fig. 4:**
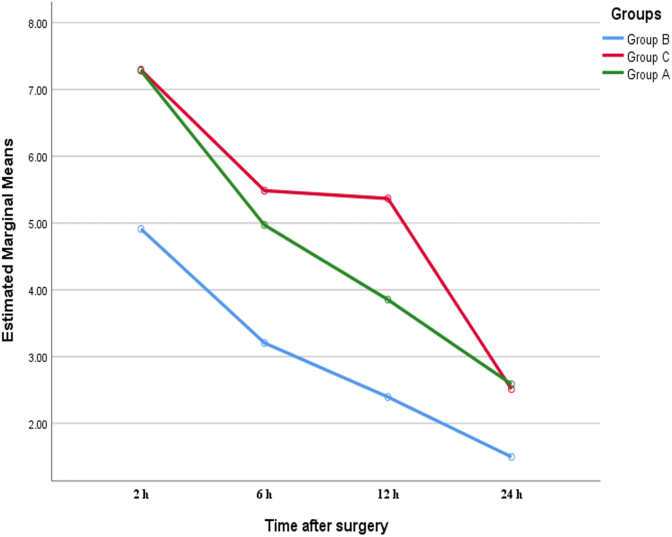
Comparison of the trend of change in mean pain intensity during the study between the study groups.

Repeated measurement of pain intensity during the study showed that in group B, the pain intensity of patients decreased significantly during the study (F (2.35–157.79) = 158.74, P < 0.001). Significant reduction of pain was also observed during the study in groups A (F (2.31–155.03) = 195.73, P < 0.001) and C (F (2.62–175.94) = 147.63, P < 0.001). However, the results of the general linear model with repeated measurements showed that in comparison between groups B and C (F (2.66–69.10) = 17.08, P < 0.001) and between groups B and A (F (2.35–29.92)) = 8.75, P < 0.001), there was a statistically significant difference between groups A and C (F (2.57–54.20) = 11.38, P < 0.001) and most of the reduction in pain intensity of patients during the study, was in groups B, A and C, respectively(Fig. 4).

### 3.1 Need of analgesics

**Fig. 5:**
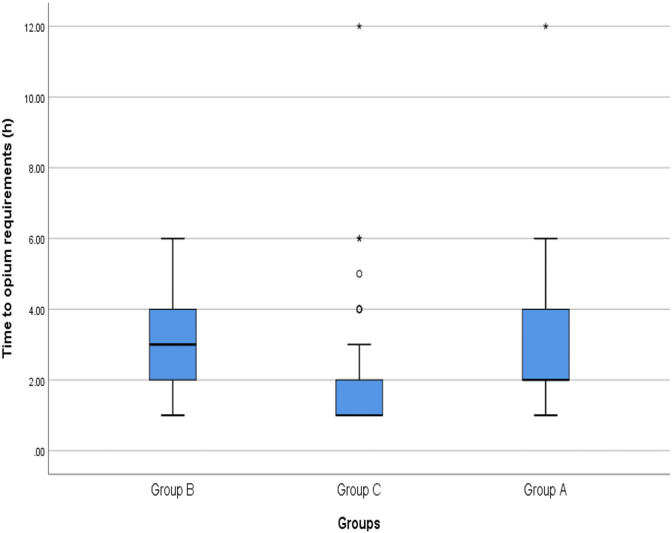
Comparison of the average time interval between surgery and the need for housing between the study groups.

Evaluation regarding the need of analgesics at different time intervals after the surgery showed that the time interval after surgery to the onset of the need for opioid analgesia in group A was 2.87 ± 2.06 h after surgery (between 1 and 12 h, Median = 2 h), group B was 3.53 ± 1.51 h after surgery (between 1 and 6 h, Median = 3 h) and in group C was 2.03 ± 1.73 h after surgery (between 1 and 12 h, Median = 1 h). The time interval between the end of surgery and the patient's need for analgesia was significantly longer in group B patients than in group A patients (P = 0.035) and C patients (P < 0.001). Also, this time interval was significantly longer in group A patients than in group C patients (P = 0.011)(Fig. 5).

**Fig. 6:**
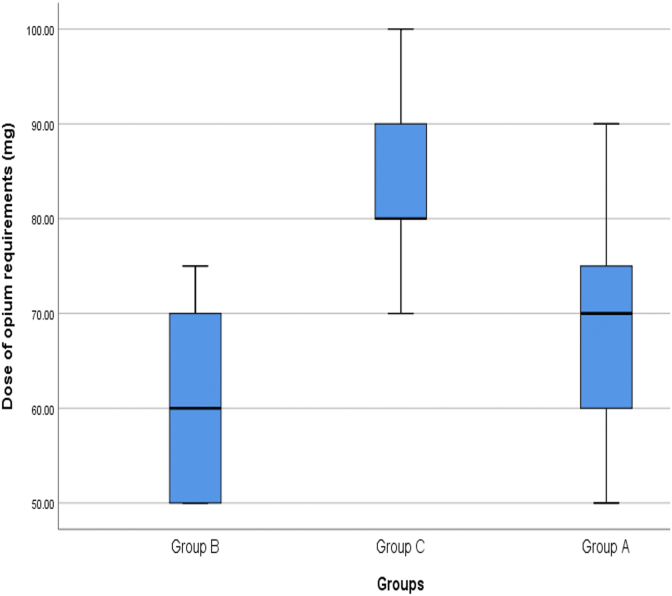
Comparison of the average opium dose of patients in the study groups after surgery.

The mean dose of opioid in patients in group A was 70.37 ± 10.09 mg (between 50 and 90 mg, Median = 70 mg), in group B was 59.12 ± 9.22 mg (between 50 and 75 mg, Median = 60 mg) and in patients in Group C was 84.34 ± 9.88 mg (between 70 and 100 mg, Median = 80 mg). The mean dose in group B was significantly lower than patients in group A (P < 0.001) and C (P < 0.001). The mean dose in group A and group C (P < 0.001) (Fig. 6).

### 3.2 Comparison of the time of onset of physical activity of the patient

**Fig. 7:**
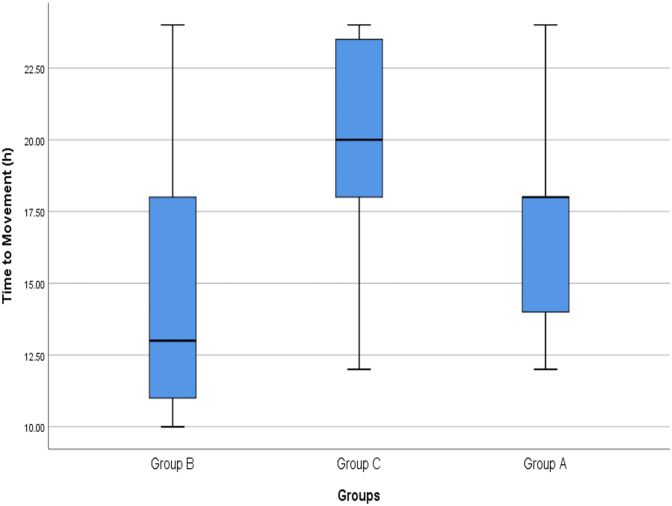
Comparison of the mean time interval between surgery and movement of patients in the study groups.

The mean time for movement of patients in group A patients was 16.94 ± 3.47 h after surgery (between 12 and 24 h, Median = 18 h) in group B was 14.66 ± 4.17 h (between 10 and 24 h, Median = 13 h) and in group C was 19.51 ± 3.70 h after surgery (between 12 and 24 h, Median = 20 h). The mean time interval between surgery and resumption of physical movement of patients in group B patients was significantly shorter than patients in groups A (P = 0.001) and C (P < 0.001). This time interval was also significantly shorter in group A patients than in group C (P < 0.001) (Fig. 7).

### 3.3 Side effects of melatonin

A total of 14 patients were presented with headache and nausea/vomiting. 7 patients (10.7%) were in group A and 7 patients (10.7%) were in group C. None of the patients in group B reported such side effects. The incidence of complications was significantly lesser in group B patients from group C patients (RR: 2.11, 95% CI: 1.76–2.54, P = 0.007) and group A (RR: 2.11, 95% CI: 1.76–2.54, P = 0.007). However, there was no statistically significant difference in the incidence of complications between groups A and C (P = 1).

## 4 Discussion

The mean pain intensity after surgery in patients receiving 5 mg melatonin was significantly lower than in the placebo group and in patients receiving 10 mg melatonin. Similar outcomes were seen in terms of time interval between the end of surgery and the need of analgesia, favoring 5 mg melatonin. The mean dose of opioid required was least with 10 mg melatonin, followed by 5 mg melatonin and placebo group, showing that melatonin is likely to be associated with dose-dependent decrease in the need of opioid following cesarean section [29]. The mean time interval between surgery and resumption of the physical activity was also least with high dose of melatonin (10 mg) relative to two other groups [30]. Side effects were insignificantly correlated in all three groups.

Melatonin is a hormone that is primarily secreted by the pineal gland and plays an important role in regulating the body's circadian rhythm. Melatonin has various effects such as effect, anti-anxiety, antioxidant, analgesic and sedative 4–6. However, data validating analgesic effects of melatonin is still not validated.

In a randomized double-blind clinical study, Vidor, et al. [31] reported that 5 mg of melatonin for four weeks is effective for treating myofascial temporomandibular disorder. These findings were significant relative to the placebo group. It improves the quality of sleep and reduces the requirement of other analgesics throughout the study period. Caumo et al. [32] evaluated preoperative administration of 5 mg melatonin in 33 patients undergoing abdominal hysterectomy in a double-blind placebo-controlled study. The results of the study showed that patients in melatonin group were presented with reduced need of patient-controlled analgesia and reduced postoperative pain following 24 h after the surgery. In a randomized clinical trial conducted on 52 patients, Borazan et al., reported that administration of 6 mg oral melatonin tablet overnight and 1 h before surgery is associated with reduced requirement of tramadol following 6, 12, 18 and 24 after the surgery and corresponding reduced postoperative pain, compared to placebo. The sedation was higher in melatonin group at 1 and 2 postoperative hours, respectively [33]. In a prospective randomized double-blind study by Khezri et al. [34], 120 patients undergoing cesarean section received 3 mg, 6 mg or placebo, 20 min prior to spinal anesthesia. The study reported that the time at first dose of analgesia was required was not significantly different in the three groups following 24 h after the surgery, however, patients who revived 3 mg of melatonin required less analgesia compared to the other two groups. In the present study, patients receiving melatonin needed a lower dose of analgesia, and this decrease in analgesic dose increased with increasing melatonin dose. The finding of our study seems more logical considering that the analgesic effect of melatonin is dose-dependent and increases with increasing dose [[35], [36], [37]].

However, there are other studies that have reported conflicting results, stating that melatonin cannot reduce the severity of pain and need for other analgesics. A double-blind, placebo-controlled study by Naguib and Samarkandi was conducted on 75 patients received who a single dose of 5 mg melatonin 100 min before laparoscopic gynecological surgery. Postoperative 15, 30, 60 and 90 min did not show any significant reduction in the pain and the amount of analgesia required [26].

In the study of Khezri et al. [34], the incidence of complications was almost the same in the three groups, and only in the group receiving 6 mg melatonin, the incidence of headache was significantly higher than the other two groups. However, in the present study, no side effects were observed in patients receiving 10 mg melatonin at a dose of 10 mg, while in patients receiving placebo and 5 mg melatonin were presented with headache and nausea/vomiting. Headache and nausea/vomiting do not seem to be related to melatonin and could be more related to the amount of analgesic drug received due to the severity of pain in patients [38].

In the present study, patients in the placebo group and the melatonin group received more opioid analgesia at a dose of 5 mg. Similarly, in the study of Khezri et al. [34], patients receiving melatonin at a dose of 6 mg received more analgesia than patients receiving melatonin at a dose of 3 mg. On the other hand, studies have shown that the incidence of side effects of prescribed exogenous melatonin, even at high intravenous doses, is very rare [39,40]. A review reported long-term side effects of melatonin like dizziness, drowsiness and somnolence in patients undergoing general anesthesia surgery [41] and cognitive decline in elderly patients under general anesthesia in hip surgery [42,43].

Our study does not the report comparison with lower dose of melatonin and other preoperative analgesics. Furthermore, intraoperative parameters can act as confounding variables in determining the intensity of postoperative pain. Since, data from such studies are based on patients’ perception of pain, studies with larger sample size are required.

## 5 Conclusion

The results of the present study showed that the use of 10 mg of melatonin before cesarean section with spinal anesthesia is not only safe, but also reduces the severity of patients' pain, increases the duration of postoperative analgesia, reduces the need for analgesics after surgery and time of patients’ ability to resume physical activity is shorter.

## Provenance and peer review

Not commissioned, externally peer-reviewed.

## Conflict of interest

The authors deny any conflict of interest in any terms or by any means during the study.

## Human and animal rights

No animals were used in this research. All human research procedures followed were in accordance with the ethical standards of the committee responsible for human experimentation (institutional and national), and with the Helsinki Declaration of 1975, as revised in 2013.

## Consent for publication

Informed consent was obtained from each participant.

## Availability of data and materials

All relevant data and materials are provided with in manuscript.

## Funding

None.

## Contributors’ statement page

Dr. Farshad Hassanzadeh Kiabi and Dr. Goli Aezzi: conceptualized and designed the study, drafted the initial manuscript, and reviewed and revised the manuscript.

Dr. Seyed Abdollah Emadi and Dr.Negar Shahzadeh Ahmadi: Designed the data collection instruments, collected data, carried out the initial analyses, and reviewed and revised the manuscript.

Dr. Aghdas Ebadi Jamkhaneh: Coordinated and supervised data collection, and critically reviewed the manuscript for important intellectual content.

Tween surgery and movement of patients in the study groups.

## Declaration of competing interest

**Approval of the research protocol:** N/A **Informed Consent:** Informed consent was obtained from each participant.
